# Smad3 Sensitizes Hepatocelluar Carcinoma Cells to Cisplatin by Repressing Phosphorylation of AKT

**DOI:** 10.3390/ijms17040610

**Published:** 2016-04-22

**Authors:** Hong-Hao Zhou, Lin Chen, Hui-Fang Liang, Guang-Zhen Li, Bi-Xiang Zhang, Xiao-Ping Chen

**Affiliations:** Hepatic Surgery Centre, Tongji Hospital, Tongji Medical College, Huazhong University of Science and Technology, 1095 Jiefang Avenue, Wuhan 430030, Hubei, China; zhouhonghao2008@163.com (H.-H.Z.); chenlin_tj@126.com (L.C.); lianghuifang1997@126.com (H.-F.L.); bruceleehust@163.com (G.-Z.L.)

**Keywords:** hepatocelluar carcinoma, smad3, AKT, drug resistance, cisplatin, LY294002

## Abstract

Background: Heptocelluar carcinoma (HCC) is insensitive to chemotherapy due to limited bioavailability and acquired drug resistance. Smad3 plays dual roles by inhibiting cell growth initially and promoting the progression of advanced tumors in HCC. However, the role of smad3 in chemosensitivity of HCC remains elusive. Methods: The role of smad3 in chemosensitivity of HCC was measured by cell viability, apoptosis, plate colony formation assays and xenograft tumor models. Non-smad signaling was detected by Western blotting to search for the underlying mechanisms. Results: Smad3 enhanced the chemosensitivity of HCC cells to cisplatin. Smad3 upregulated p21^Waf1/Cip1^ and downregulated c-myc and bcl2 with the treatment of cisplatin. Moreover, overexpression of smad3 repressed the phosphorylation of AKT, and *vice versa*. Inhibition of PI3K/AKT pathway by LY294002 restored chemosensitivity of smad3-deficiency cells to cisplatin in HCC. Conclusion: Smad3 sensitizes HCC cells to the effects of cisplatin by repressing phosphorylation of AKT and combination of inhibitor of AKT pathway and conventional chemotherapy may be a potential way to solve drug resistance in HCC.

## 1. Introduction

Hepatocellular carcinoma (HCC) is the sixth most common malignant tumor and the second leading cause of cancer-related death worldwide [1,2]. Surgical resection is currently well-accepted treatment for early HCC, and five-year overall survival rate is up to 60% [2,3]. However, five-year cumulative recurrence rate is 80% to 100% [4,5]. In this circumstance, chemotherapy may be the potent approach to remove the residual tumor cells and reduce HCC recurrence. Until now, chemotherapy has limited effects because of limited bioavailability and acquired drug resistance in HCC [6]. Furthermore, the mechanisms of drug resistance in HCC are complex and elusive.

Transforming growth factor beta (TGF-β) is a potent pleiotropic cytokine that regulates cell proliferation, adhesion, migration, and apoptosis [7]. It plays diverse and conflicting roles in HCC, acting as a tumor-suppressor in early carcinogenesis but enhancing tumor dissemination at later stages [8]. These processes may be mediated through smad-dependent and smad-independent pathways [9]. The smad-dependent pathway, also called the canonical smad pathway, phosphorylates smad2 and smad3 by TGFBR1, then hetero-oligomerize with smad4 and translocate to the nucleus to mediate target gene expression or repression [10]. The smad-independent pathways contain p38 mitogen-activated protein kinase (MAPK), p42/p44 MAPK, Jun N-terminal kinase (JNK) MAPK, phosphatidylinositol 3-kinase (PI3K)/AKT, *etc*. They can be activated not only by TGF-β, but also by a variety of extracelluar stimuli such as insulin and chemotherapeutics [11]. Once activated, they further phosphorylate and activate cytoplasmic proteins transmitting the signal from cytoplasm to nucleus. They can directly regulate the function of various cellular processes by controlling different target genes. Interestingly, smad and non-smad pathways can interact with each other and regulate cell proliferation, apoptosis, migration, drug resistance, *etc.* [11].

*Cis*-dichlorodiamineplatinum(II) (cisplatin) was the first platinum anti-cancer drug in clinical treatment. Currently, it is one of the most effective and commonly used chemotherapeutics in many solid tumors [12]. It is often used by implantable chemotherapy pump or tanscatheter arterial chemoembolization (TACE) in HCC. However, its effect is not ideal due to limited bioavailability and acquired drug resistance. The underlying mechanisms contained alterations of MAPK and PI3K/AKT pathways [13], dysfunction of tumor suppressor genes (like p53) [14] and oncogenes (like c-Fos, c-Jun) [15,16], alterations in expression of pro- and anti-apoptotic proteins (like Bcl-2 and c-myc), *etc.* [17,18].

Previous studies showed that smad3 played different roles in modulating chemoresponses that is context-dependent and non-smad pathways also played vital roles in this process [9,19]. In the present study, we aimed to profile and characterize the effects of smad3 in chemosensitivity of HCC to cisplatin and identify potential links between smad3 and non-smad signaling in mediating cisplatin sensitivity.

## 2. Results

### 2.1. Smad3 Increases the Sensitivity of Heptocelluar Carcinoma (HCC) to Cisplatin in Vitro

**Figure 1:**
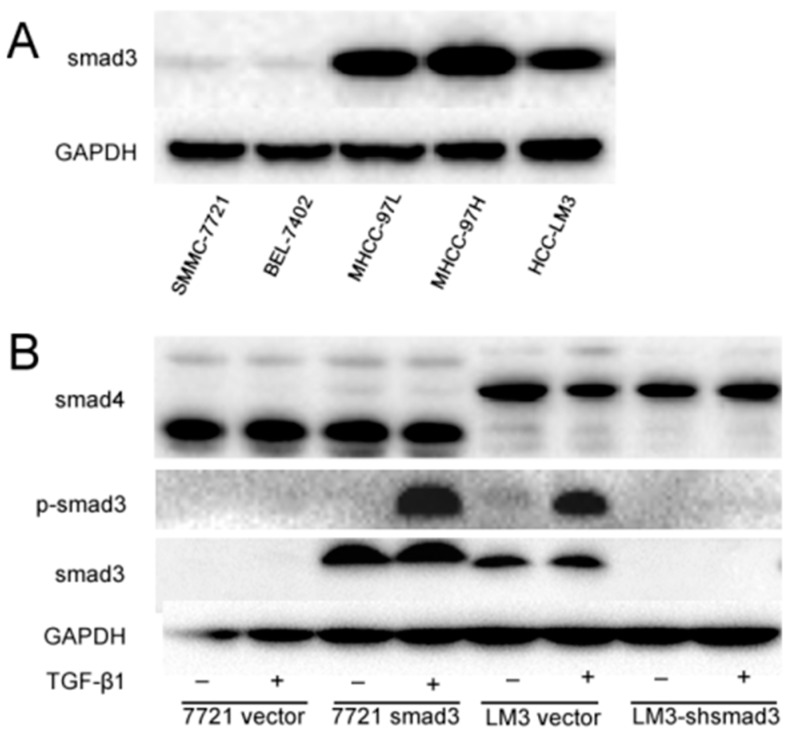
Transforming growth factor-β (TGF-β) signaling is intact in smad3-expressing heptocelluar carcinoma (HCC) cells. (**A**) The expression of smad3 in HCC cell lines was detected by Western blot; (**B**) Stable overexpression of smad3 in SMMC-7721 cells and knockdown of smad3 in HCC-LM3 cells were confirmed by Western blot. Smad3 phosphorylation was activated upon TGF-β treatment in smad3-expressing cells. All of the experiments were performed three times and representative pictures are shown.

To investigate the role of smad3 in HCC drug sensitivity, we detected the expression of smad3 in HCC lines (Figure 1A). We chose SMMC-7721 and HCC-LM3 cells as smad3-deficiency and smad3-expressing representatives, respectively. Then, we knocked in smad3 in SMMC-7721 cells and knocked down smad3 in HCC-LM3 cells as previously described [20]. We also detected TGF-βsignaling cascade in these two pair cells and we observed that canonical TGF-β signaling was intact and similarly activated by its ligand, as shown by smad3 phophorylation with the treatment of TGF-β1 in smad3-expressing cells (Figure 1B).

**Figure 2:**
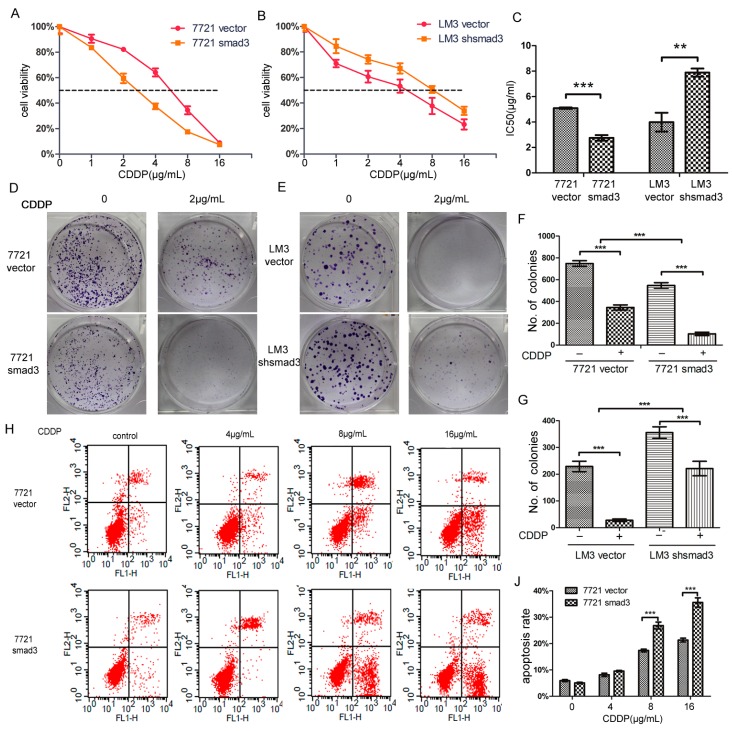

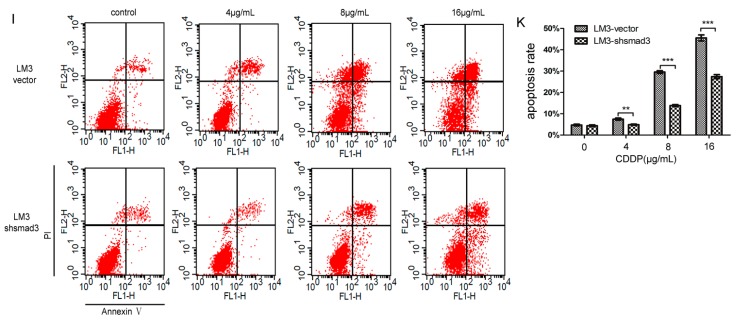
Smad3 increases the sensitivity of HCC to cisplatin *in vitro*. SMMC-7721 (**A**) and HCC-LM3 (**B**) cells were treated with indicated concentrations of cisplatin for 48 h. The number of viable cells was determined by CCK-8. Relative percentages of live cells were analyzed by comparing with cells without cisplatin treatment; (**C**) The 50% inhibitionary concentration values (IC_50_) of 7721 and LM3 cells were calculated and analyzed by Graphpad Prism 5.0; (**D**–**G**) Plate colony formation assay was performed to detect cisplatin sensitivity, and the number of colonies was evaluated 14 days after cell plating. The data are presented as the mean ± S.D. from three wells; (**H**–**K**) Cell apoptosis assays were examined using Fluorescence activated cell sorting (FACS) 48 h after cisplatin treatment. The percentages of apoptotic cells are presented as the mean ± S.D. from three wells (** *p* < 0.01, *** *p* < 0.001).

We treated SMMC-7721 and HCC-LM3 cells with cisplatin, and performed CCK-8 assay. 7721-smad3 cells showed higher sensitivity to cisplatin compared with its control 7721-vector cells (Figure 2A). Meanwhile, LM3-vector and LM3-shsmad3 cells showed the same effects to cisplain (Figure 2B). The 50% inhibitory concentration (IC_50_)values of 7721-vector and 7721-smad3 cells were 3.822 ± 0.095 and 2.062 ± 0.080 ng/mL, respectively, and the differences were statistically significant (*p* < 0.001) (Figure 2C). Similarly, the IC_50_ values of LM3-vector and LM3-shsmad3 cells were 2.781 ± 0.053 and 4.579 ± 0.262 ng/mL, respectively, which also had significant differences (*p* < 0.005) (Figure 2C). To further verify this phenomenon, we performed plate cloning formation assays. Although the number of colonies in 7721-smad3 and LM3-vector cells were less than 7721-vector and LM3-shsmad3 cells, respectively, the colony formation efficiency decreased more in smad3 over-expression cell lines than in smad3-deficient cell lines in the presence of cisplatin (2 µg/mL) (Figure 2D–G). We also conducted AnnexinV-FITC flow cytometry to evaluate apoptotic rate of HCC cells with the treatment of cisplatin. In SMMC-7721 cells, cisplatin increased apoptosis by 15.4% ± 1.3% in 7721-vector cells and by 30.5% ± 2.4% in 7721-smad3 cells (Figure 2H–J). Synchronously, the apoptotic rate in LM3-vector cells was increased by 29.6% ± 3.1%, while in LM3-shsmad3 cells, by 13.8% ± 3.5% (Figure 2I–K). The differences were statistically significant (*p* < 0.001). Taken together, these results indicated that smad3 sensitized HCC cells to cisplatin.

### 2.2. Smad3 Increases the Sensitivity of HCC to Cisplatin in Vivo

**Figure 3:**
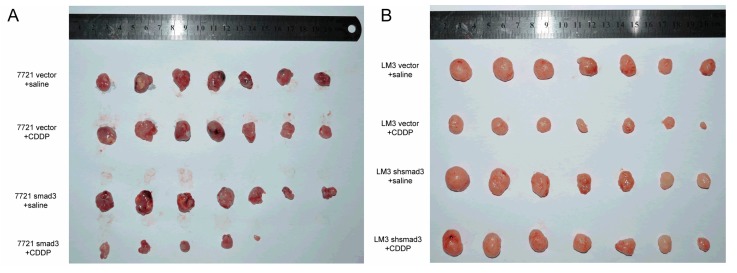

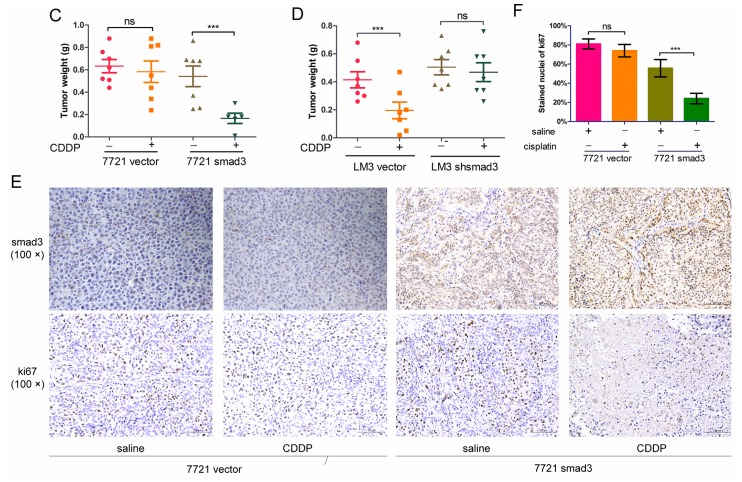
Smad3 increases the sensitivity of HCC to cisplatin *in vivo*. SMMC-7721 (**A**) and HCC-LM3 (**B**) cells were injected subcutaneously into Balb/c-nu mice and cisplatin was injected i.p. every three days; (**C**,**D**) Tumor weight was examined after mice were sacrificed; (**E**) Subcutaneous tumors were subjected to smad3 and ki67 staining. Representative pictures are shown (100×); (**F**) The percentage of ki67 stained nuclei was calculated in different groups. All the results are represented as the mean ± S.D. from three independent trials. (*** *p* < 0.001; n.s. means no significance).

To confirm the effects of smad3 and cisplatin *in vivo*, we established subcutaneous xenograft model by injecting smad3-expressing and smad3-deficient HCC cells into Balb/c-nude mice. Intra-peritoneal cisplatin treatment was initiated when the tumor volume reached approximately 100 mm^3^ on day 7, and mice were sacrificed on day 28. Treatment with cisplatin (2 mg/kg) had no significant effect on the growth of 7721-vector xenograft tumors, whereas 7721-smad3 group showed a significant reduction in tumor volume and weight (Figure 3A,C). Similar results were observed in LM3-vector and LM3-shsmad3 groups with the treatment of cisplatin (Figure 3B,D). Ki67 and smad3 staining of tumor sections were performed to detect the effects of cisplatin *in vivo* (Figure 3E). Treatment with cisplatin significantly decreased the proliferation index in smad3 high-expression group, as determined by the percentage of ki67 positive cells, whereas no significant differences in smad3-deficiency group (Figure 3F).

### 2.3. Smad3 Activates MAPK but Represses AKT Signaling

**Figure 4:**
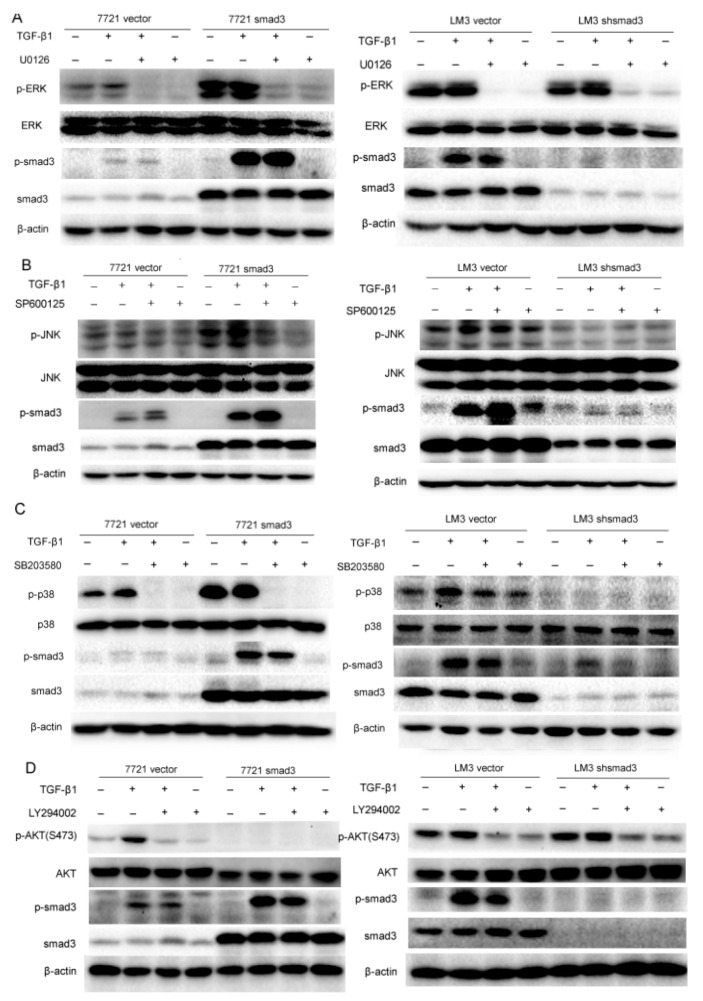
Smad3 activates mitogen-activated protein kinases (MAPK) but represses AKT signaling: (**A**) Extracelluar signal regulated kinase (ERK) and smad3 signaling were examined with the treatment of TGF-β1 (5 ng/mL, 0.5 h) and U0126 (10 µM) in 7721 and LM3 cells; (**B**) JNK and smad3 signaling were examined with the treatment of TGF-β1 (5 ng/mL, 6 h) and SP600125 (30 µM) in 7721 and LM3 cells; (**C**) P38 and smad3 signaling were examined with the treatment of TGF-β1 (5 ng/mL, 1 h) and SB203580 (30 µM) in 7721 and LM3 cells; and (**D**) AKT and smad3 signaling were examined with the treatment of TGF-β1 (5 ng/mL, 3 h) and LY294002 (20 µM) in 7721 and LM3 cells. All of the experiments were performed in triplicate and representative pictures are shown.

Since TGF-β signaling can be mediated through smad and non-smad pathways to regulate cell proliferation, invasion, metastasis, drug resistance, *etc.* [11], we investigated whether smad3 improved sensitivity of HCC cells to cisplatin through non-smad pathway. We evaluated non-smad pathways by examining the phosphorylation of ERK, JNK, p38 and AKT signaling in SMMC-7721 and HCC-LM3 cells in the presence of TGF-β1, which respresents the activation of non-smad pathway. Moreover, kinase inhibitors including U0126 (MEK1/2 inhibitor suppressed Erk signaling), SP600125 (JNK MAPK inhibitor), SB203580 (p38 MAPK inhibitor) and LY294002 (PI3K inhibitor suppressed Akt signaling) were used to evaluate the relationship of smad and non-smad pathways. The results showed that smad3 promoted activation of MAPK signaling (ERK, JNK, and p38) and repressed activation of AKT signaling in the presence of TGF-β1 (5 ng/mL). In detail, p-ERK was activated upon TGF-β1 (0.5 h) treatment and was blocked with the pretreatment of U0126. Meanwhile, U0126 did not influence the phosphorylation of smad3 (Figure 4A). The same results were observed in p38 signaling when the treatment of TGF-β1 was increased to 1 h (Figure 4B). These results indicated that ERK and p38 were just downstream effectors of smad3 but did not influence the activation of smad3. However, smad3 promoted activation of JNK in the presence of TGF-β1 (6 h) and inhibition of JNK pathway by SP600125 increased the phosphorylation of smad3, which displayed that JNK pathway was a negative feedback to inhibit the phosphorylation of smad3 (Figure 4C). Interestingly, smad3 repressed the activation of AKT with the treatment of TGF-β1 (3 h) and smad3 knock-out reversed this phenomenon. Inhibition of AKT pathway by LY294002 repressed the phosphorylation of smad3, which demonstrated that AKT and smad3 formed a positive feedback loop (Figure 4D). These results indicated that smad3 activated MAPK but repressed AKT pathway in the presence of TGF-β, and smad3 may sensitize HCC cells to cisplatin by blockage of AKT pathway.

### 2.4. Smad3 Represses AKT Phosphorylation and Regulates Apoptosis-Related Proteins in the Presence of Cisplatin

**Figure 5:**
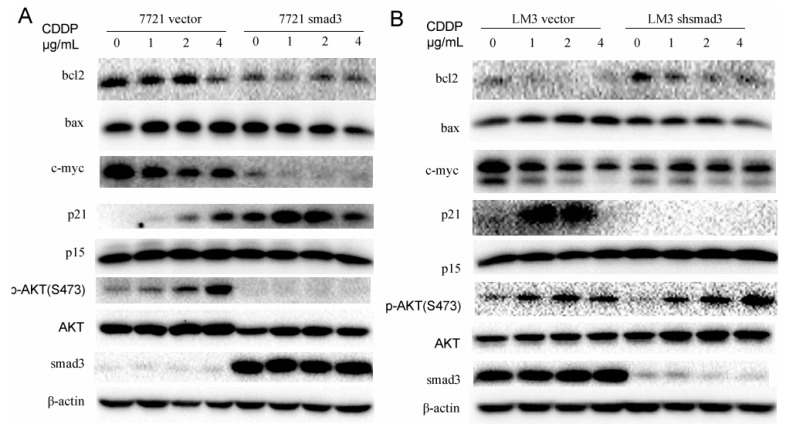
Smad3 represses AKT phosphorylation and regulates apoptosis-related proteins in the presence of cisplatin. (**A**,**B**) Western blot assay was performed using lysates from SMMC-7721 and HCC-LM3 cells after treating with cisplatin (48 h) for different concentration points. The expression of p15, p21, c-myc, bcl2, bax, p-AKT (S473), AKT, and smad3 were examined; β-actin was used as a loading control. All of the experiments were performed in triplicate and representative pictures are shown.

We have proven that smad3 inhibited the activation of AKT in the presence of TGF-β1. To investigate whether AKT pathway plays an important role in the drug resistance of cisplatin, we treated 7721 and LM3 cells with cisplatin and performed Western blot assay to verify it. Smad3 over-expression in 7721 cells inhibited the activation of AKT with the treatment of cisplatin. Meanwhile, smad3 downregulation in HCC-LM3 cells increased the phosphorylation of AKT. These results indicated that smad3-deficiency cells might induce drug resistance by activation of AKT pathway. Downstream transcriptional factors of smad3 and AKT and some apoptosis-related proteins were also detected in the presence of cisplatin. P21^Waf1/Cip1^, a transcription factor of smad3 and a potent cyclin-dependent kinase inhibitor, was activated in 7721-smad3 cells to induce cell apoptosis in the presence of cisplatin. Meanwhile, C-myc expression was decreased to disappear in 7721-smad3 cells compared with 7721-vector cells. Bcl2, an important member of mitochondria apoptosis, was also decreased after cisplatin treatment in 7721-smad3 cells. However, the expression of bax and p15 did not change in the presence of cisplatin, which indicated that they did not participate in this process. The drug was also used in LM3-vector and LM3-shsmad3 cells and similar effects were observed (Figure 5B). These results indicated that smad3 repressed AKT phosphorylation and regulated apoptosis-related proteins to sensitize HCC cells to cisplatin.

### 2.5. LY294002 Restores Chemosensitivity of HCC in Smad3-Defeciency Cells

**Figure 6:**
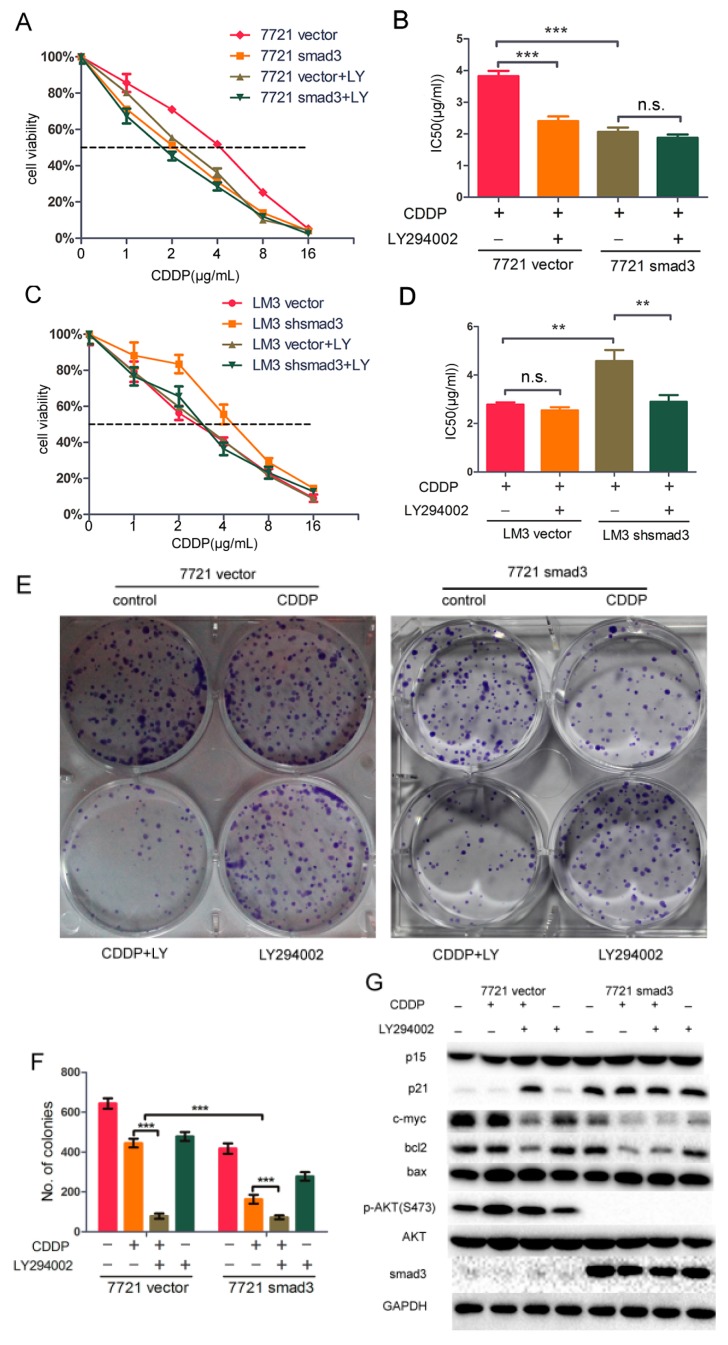
LY294002 restores chemosensitivity of HCC in smad3-defeciency cells. (**A**–**D**) SMMC-7721 and HCC-LM3 cells were treated with indicated concentrations of cisplatin with or without LY294002 for 72 h. The number of viable cells was determined by CCK-8. IC_50_ values were calculated and analyzed by Graphpad Prism 5.0. Data are presented as mean ± S.D. from triplicate wells; (**E**,**F**) Plate colony formation assay was performed to detect the synergistic effects of cisplatin and LY294002, and the number of colonies was evaluated 14 days after cell plating. The data are presented as the mean ± S.D. from three wells; (**G**) SMMC-7721 cells were treated with cisplatin and/or LY294002 for 48 h and cell lysates were used for Western blot assays. All of the experiments were performed in triplicate (** *p* < 0.01, *** *p* < 0.001; n.s. means no significance).

In this study, we demonstrated that smad3 sensitized HCC cells to cisplatin and inhibited phosphorylation of AKT pathway, and we hypothesized that AKT pathway induced drug resistance of cisplatin in smad3-deficiency cells. To verify this hypothesis, we treated SMMC-7721 and HCC-LM3 cells with cisplatin and/or AKT inhibitor LY294002. The smad3-deficiency cells showed significantly higher sensitivity to cisplatin in the presence of LY294002 (Figure 6A,C). The IC_50_ value of cisplatin decreased from 3.822 ± 0.095 to 2.400 ± 0.088 ng/mL in 7721-vector cells with the treatment of LY294002, whereas the value decreased from 2.062 ± 0.080 to 1.876 ± 0.060 ng/mL in 7721-smad3 cells (Figure 6B). Meanwhile, The IC_50_ value of cisplatin decreased from 4.579 ± 0.262 to 2.899 ± 0.159 ng/mL in LM3-vector cells with the treatment of LY294002, and from 2.781 ± 0.053 to 2.541 ± 0.077 ng/mL in LM3-shsmad3 cells (Figure 6D). Plate clone formation assays were also performed to prove this hypothesis. The combination of cisplatin and LY294002 significantly inhibited colony formation in 7721-vector cells compared with cisplatin or LY294002 alone (Figure 6E,F). Other MAPK inhibitors were also used with cisplatin together, but the results were not ideal compared with LY294002 (data not shown). These results suggested that combination of LY294002 and cisplatin sensitized the smad3-deficiency cells up to the same extent as cisplatin alone did in smad3-expressing cells.

LY294002 not only inhibited the phosphorylation of AKT, but also regulated cell-proliferation and cell cycle proteins to sensitize smad3-deficient cells to cisplatin. The expression of p21 in 7721-vector with the treatment of cisplatin and LY294002 increased to a similar level as that in 7721-smad3 cells with the treatment of cisplatin. The expression of bcl2 and c-myc was positively correlated with the activation of AKT. Meanwhile, the expression of p15 and bax did not change significantly, which was consistent with our previous study (Figure 6G). Taken together, these results indicated that LY294002 restored cisplatin sensitivity in smad3-deficiency cells through the repression of AKT pathway.

## 3. Discussion

Due to mild symptoms at early stage in HCC, almost half of the patients are diagnosed at advanced stage and not suitable for surgery [21]. Meanwhile, high recurrence after operation due to residual tumor cells also leads to unfavorable prognosis [3]. For patients with advanced stage, sorafenib is currently the only targeted drug approved by FDA for HCC, which provides a survival advantage of 2.8 months in selected patients [22]. Chemotherapy has been widely regarded as ineffective due to toxicity and limited response in HCC [6]. Thus, the improvements of understanding underlying mechanisms of drug resistance are urgent needed.

Smad3 functions as a transcriptional modulator activated by transforming growth factor-beta and plays dual roles as tumor suppressor and tumor promoter in HCC, which is context-specific [23]. Smad3 mediates TGF-β induced growth inhibition through down-regulation of c-myc [24] and induction of p15^Ink4b^ [25] and p21^Cip1^ [26]. It reduces susceptibility to HCC by repressing bcl-2 transcription in a chemically induced murine model [27]. However, smad3 promotes tumor progression at advanced stages by suppressing immune surveillance, inducing EMT and enhancing pro-metastatic transcription factors such as snail and slug [28,29,30,31]. Moreover, the role of smad3 can shift from tumor suppressor to tumor promoter by regulating its phospho-isoforms [9,32]. However, the role of smad3 in chemosensitivity of HCC remains elusive.

In the present study, we have identified smad3 sensitized HCC cells to cisplatin *in vitro* and *in vivo*. Cisplatin is currently still used in HCC by chemotherapy pump and trans-arterial chemoembolization, despite its effect is uncertain due to lacking of evidences. Our findings indicate that smad3 may be a biomarker to identify whether the patients are suitable for cisplatin as an adjuvant treatment. Nonetheless, the different phospho-isoforms of smad3 should be considered and the expression of smad3 should also be detected in cisplatin-sensitive and insensitive patients to further confirm our conclusion. In our study, smad3 promoted HCC cells apoptosis by induction of p21 and repression of c-myc and bcl2 with the treatment of cisplatin. This is consistent with other studies [24,25,26,27]. TGF-β mediated transcriptional repression of c-myc is dependent on direct binding of smad3. Bcl2 and p21 are all common targets of TGF-β signaling, the alteration of these genes further confirmed that smad3 sensitized HCC cells to cisplatin.

AKT, one of the most frequently hyper-activated signaling in human cancers, plays an important role in both carcinogenesis and chemo-resistance [33,34]. Significant correlation between activation of AKT and poor prognosis suggests an important role of AKT activation in HCC [35]. Over-expression of myr-AKT1alone leads to liver tumor development after 6 months [36]. Cisplatin activates PI3K/AKT signaling and results in cisplatin resistance in ovarian cancer [37]. Here, we found that AKT phosphorylation was activated when HCC cells were exposure to cisplatin. However, when smad3 expression was overexpressed in SMMC-7721 cells, the phosphorylation of AKT was blocked totally. Consistently, when smad3 expression was reduced in HCC-LM3 cells, the phosphorylation of AKT was increased compared with its control cells upon cisplatin treatment. Meanwhile, 7721-smad3 and LM3-vector cells were more sensitive to cisplatin compared with their smad3-deficiency cells, respectively. These results suggested that smad3 sensitized HCC cells to cisplatin by repressing AKT pathway.

We also observed that LY294002, an inhibitor of PI3K/AKT pathway, restored chemosensitivity in smad3-deficiency cells to cisplatin. Targeted inhibition of AKT pathway shows promise in the treatment of HCC given its role in carcinogenesis and drug resistance. Until now, several small inhibitors of AKT have been developed and are in clinical trials. For example, MK2206, a potent oral pan-AKT inhibitor, is investigated in several phase I and phase II clinical trials [38,39]. Treatment with MK-2206 alone safely results in significant AKT pathway blockade in patients with advanced solid tumors [38]. Another clinical trial shows that MK-2206 plus carboplatin and paclitaxel, docetaxel, or erlotinib is well-tolerated, with early evidence of antitumor activity [39]. We have identified that LY294002 overcame drug resistance of smad3-defeciency cells to cisplatin and combination of LY294002 and cisplatin can effectively induced cancer cells apoptosis in HCC. The result is consistent with ongoing clinical trials, even though our experiments did not use the latest agents. Currently, transcatheter arterial chemoembolization (TACE) and transarterial infusion chemotherapy (TAI) are widely used for patients with unresectable or recurrent HCC of any Child–Pugh class [40]. However, the objective response rate of cisplatin is only 17% when used as monotherapy for HCC [41]. Thus, the AKT inhibitor alone or in combination with conventional chemotherapeutics or targeted drugs should be further investigated in clinical trials as potential novel therapeutic agents in HCC.

## 4. Methods and Materials

### 4.1. Cell Lines and Culture

Human hepatocarcinoma cell lines SMMC-7721 and BEL-7402 were purchased from China Center for Type Culture Collection (CCTCC, Wuhan, China). MHCC-97L, MHCC-97H, and HCC-LM3 was obtained from Liver Cancer Institute, Zhongshan Hospital, Fudan University, Shanghai, China. All of the above HCC cell lines were maintained in Dulbecco modified Eagle medium (DMEM) supplemented with 10% fetal bovine serum (FBS).

### 4.2. Materials

Cisplatin and TGF-β1 (Sigma-Aldrich, St. Louis, MO, USA) were dissolved in PBS and 10 mM Citric Acid (pH 3.0), respectively. Small inhibitors LY294002, SB203580, U0126, SP600125 (SelleckChemicals, Houston, TX, USA) were dissolved in DMSO. Phospho-Akt (Ser473), phospho-smad3 (Ser423/425), phospho-ERK (Thr202/Tyr204), Erk, phospho-p38 (Thr180/Tyr182), SAPK/JNK, phospho-SAPK/JNK (Thr183/Tyr185), p15^INK4B^, p21^Waf1/Cip1^, bax, bcl2, and ki67 were purchased from Cell Signaling Technology (Beverly, MA, USA). P38, pan-Akt, and β-Actin were purchased from Santa Cruz Biotechnology Inc (Santa Cruz, CA, USA). Smad3 and c-myc were purchased from Abcam Co Ltd (Cambridge, UK). Horse radish peroxidase (HRP) conjugated secondary anti-rabbit and antimouse IgG antibodies were from Sigma-Aldrich Co., Ltd.

### 4.3. Retrovirus Production, Virus Infection and Establishment of Stable Cell Clones

LPCX Smad3 was a gift from Rik Derynck (Addgene plasmid # 12638) [42] and pRetroSuper Smad3 was a gift from Joan Massague (Addgene plasmid # 15726) [43]. Retroviral supernatants were produced by transfection of 293T cells by Lipofectamine 2000 (Life Technologies, Carlsbad, CA, USA). Transfected 293T cells were used to collect retrovirus-containing supernatants 48 h after transfection. Collected retroviral supernatants were filtered through a 0.45 μm filter and were transfected into the HCC cells with a multiplicity of infection (MOI) ranging from 30 to 50 in the presence of polybrene (8 µg/mL). Twenty-four hours after infection, cells were selected for 2 weeks using puromycin (5 µg/mL). 7721 vector, 7721 smad3, LM3-vector, LM3-shsmad3 cells were generated and used for the following experiments.

### 4.4. Cell Viability Assay

SMMC-7721 and HCC-LM3 cells (5 × 10^3^) were counted by Cellometer Mini (Nexcelom Bioscience, Lawrence, MA, USA) and plated in 96-well plates, then incubated overnight. Cells were treated with indicated concentrations of cisplatin with or without LY294002 for 48 and 72 h. The number of viable cells was determined by CCK-8 following the kit protocol. The cell viability was calculated as (OD_450_ value of drug-treatment)/(OD_450_ value of control group) × 100%.

### 4.5. Colony Formation Assay

SMMC-7721 (5 × 10^2^) and HCC-LM3 cells (3 × 10^3^) were plated in 6-well plates and incubated overnight. Cells were treated with indicated concentrations of cisplatin and (or) LY294002. 10 days later, cells were stained with crystal violet. The colony pictures were taken with a Nikon DSLR Camera.

### 4.6. Flow-Cytometric Analysis

SMMC-7721 and HCC-LM3 cells (1 × 10^5^) were plated in 6-well plates and incubated overnight. Cells were treated with indicated concentrations of cisplatin for 72 h. Cells were harvested and detected with Annexin V-FITC/PI apoptosis detection Kit (BD bioscienses, San Jose, CA, USA).

### 4.7. Tumorigenicity Assay

Six-week-old male BALB/c-nu mice were used for experiments. All of the animal studies met the National Institutes of Health guidelines (NIH publication 86–23 revised 1985) and were approved by the Committee on the Ethics of Animal Experiments of Tongji Medical College, HUST.

For *in vivo* tumorigenicity assays, 1 × 10^6^ 7721-vector, 7721-smad3, LM3-vector, and LM3-shsmad3 cells were injected subcutaneously into the right flank of nude mice. Cisplatin (2 mg/kg) was injected intra-peritoneal every 3 days. Mice were sacrificed on Day 28. Tumor volume and tumor weight were calculated to evaluate tumor formation ability.

### 4.8. Western Blotting Assay

Protein extracted was separated by SDS-polyacrylamide gels (SDS-PAGE) and transferred to polyvinylidene difluoride (PVDF) membrane. The membrane was probed with specific primary antibody, followed by incubation with a horseradish peroxidase HRP-conjugated anti-mouse or anti-rabbit secondary antibody. Detection was performed using ChemiDoc™ Imaging Systems (Bio-Rad Laboratories Co., Ltd., Hercules, CA, USA).

### 4.9. Statistical Analysis

The data are presented as mean ± standard deviation (S.D.) of three independent experiments. Statistical analyses were performed by Student’s *t*-test or one-way analysis of variance (ANOVA). Statistical analysis was performed with Graphpad Prism 5.0 and SPSS 13.0.A value of *p* < 0.05 was considered statistically significant.

## 5. Conclusions

In conclusion, our study demonstrates that smad3 sensitized hepatocellular carcinoma cells to the effects of cisplatin by repression of AKT phosphorylation. LY294002, a PI3K/AKT inhibitor, overcomes drug resistance of smad3-defeciency cells to cisplatin in HCC. Our study shows one feasible mechanism of drug resistance in HCC, and indicates that combination of conventional chemotherapy and targeted therapy may be a potential way to overcome drug resistance and improve the prognosis of HCC patients.

## Abbreviations

HCC, hepatocelluar carcinoma; JNK, Jun N-terminal kinase; PI3K, phosphoinositide 3-kinase; MAPK, mitogen-activated protein kinase; TACE, tanscatheter arterial chemoembolization; CCK-8, Cell Counting Kit-8; FITC, fluorescein isothiocyanate; PI, propidium iodide; HRP, Horse radish Peroxidase; PAGE, polyacrylamide gels; FACS, Fluorescence activated Cell Sorting; IC_50_, 50% inhibitory concentration; ERK, extracelluar signal regulated kinase; PVDF, polyvinylidene difluoride.
